# Supplementary material for: A drug repurposing approach reveals targetable epigenetic pathways in Plasmodium vivax hypnozoites
Source: eLife. 2025 Sep 30;13:RP98221. doi: 10.7554/eLife.98221 (PMC12483515; doi:10.7554/eLife.98221)
Supplement: Supplementary file 1. — Due to an error during library plating, some plates contained only 1 well of monensin, preventing calculation of a Z′ factor for those plates (listed as N.A.). [file elife-98221-supp1.docx]

| **ReF#** | **Hypnozoites/well** | **Schizonts/well** | **Z-factor** | **Screen Location** | **PHH Lot** | ***P. vivax* Case** |
| --- | --- | --- | --- | --- | --- | --- |
| ReF01 | 111 | 77 | N.A. | IPC | BGW | Pv847 |
| ReF02 | 49 | 16 | 0.19 | IPC | UBV | Pv595 |
| ReF03 | 21 | 14 | N.A. | SMRU | UBV | PID423955 |
| ReF04 | 55 | 20 | -0.04 | IPC | UBV | Pv593 |
| ReF05 | 87 | 61 | 0.17 | IPC | UBV | Pv602 |
| ReF06 | 27 | 33 | 0.11 | SMRU | UBV | PID432054 |
| ReF07 | 30 | 30 | -0.02 | SMRU | UBV | PID432054 |
| ReF08 | 26 | 24 | -0.03 | SMRU | UBV | PID432054 |
| ReF09 | 41 | 21 | 0.13 | IPC | UBV | Pv602 |
| ReF10 | 110 | 56 | -0.01 | IPC | BGW | Pv708 |
| ReF11 | 24 | 21 | 0.06 | SMRU | UBV | PID425583 |
| ReF12 | 49 | 21 | 0.19 | IPC | UBV | Pv602 |
| ReF13 | 37 | 52 | 0.09 | IPC | UBV | Pv603 |
| ReF14 | 28 | 45 | 0.31 | IPC | UBV | Pv603 |
| ReF15 | 69 | 84 | 0.46 | IPC | UBV | Pv603 |
| ReF16 | 63 | 73 | 0.14 | IPC | UBV | Pv603 |
| ReF17 | 58 | 28 | 0.07 | IPC | UBV | Pv606 |
| ReF18 | 54 | 34 | 0.33 | IPC | UBV | Pv606 |
| ReF19 | 43 | 21 | 0.23 | IPC | UBV | Pv606 |
| ReF20 | 22 | 10 | 0.15 | IPC | UBV | Pv608 |
| ReF21 | 54 | 46 | N.A. | IPC | BGW | Pv847 |
| ReF22 | 76 | 55 | 0.04 | IPC | UBV | Pv609 |
| ReF23 | 40 | 40 | 0.06 | SMRU | UBV | PID402389 |
| ReF24 | 45 | 27 | 0.33 | IPC | BGW | Pv849 |
| ReF25 | 112 | 72 | N.A. | IPC | UBV | Pv609 |
| ReF26 | 117 | 66 | N.A. | IPC | UBV | Pv609 |
| ReF27 | 78 | 120 | N.A. | IPC | UBV | Pv624 |
| ReF28 | 107 | 61 | 0.38 | IPC | BGW | Pv847 |
| ReF29 | 69 | 54 | 0.32 | IPC | BGW | Pv836 |
| ReF30 | 65 | 36 | N.A. | IPC | UBV | Pv623 |
| ReF31 | 131 | 64 | 0.58 | IPC | UBV | Pv611 |
| ReF32 | 81 | 51 | 0.34 | IPC | UBV | Pv609 |
| ReF33 | 111 | 105 | 0.22 | IPC | BGW | Pv708 |
| ReF34 | 103 | 107 | 0.28 | IPC | UBV | Pv624 |
| ReF35 | 77 | 47 | 0.36 | IPC | UBV | Pv609 |
| ReF36 | 59 | 43 | 0.28 | IPC | UBV | Pv609 |
| ReF38 | 115 | 68 | 0.26 | IPC | BGW | Pv836 |
| ReF39 | 84 | 18 | 0.16 | IPC | BGW | Pv838 |
| ReF40 | 90 | 20 | 0.35 | IPC | BGW | Pv846 |
| ReF41 | 95 | 22 | 0.24 | IPC | BGW | Pv846 |

**Supplement File 1**. Summary of ReFRAME plate (40 plates labelled 1-41, with 37 skipped) run metrics including average hypnozoites and schizont counts per well, Z-factor for 1 μM monensin wells, screening location (Shoklo Malaria Research Unit, Thailand, or Pasteur Institute of Cambodia) PHH lot used, and *P. vivax* patient isolate used. Due to an error during library plating, some plates contained only 1 well of monensin, preventing calculation of a Z’ factor for those plates (listed as N.A.).
