# Supplementary material for: A drug repurposing approach reveals targetable epigenetic pathways in Plasmodium vivax hypnozoites
Source: eLife. 2025 Sep 30;13:RP98221. doi: 10.7554/eLife.98221 (PMC12483515; doi:10.7554/eLife.98221)
Supplement: Supplementary file 2. — Cytotoxicity (pCC50) against PSH was measured using nuclei counts. Maduramicin is a positive control with activity against P. cynomolgi hypnozoites. [file elife-98221-supp2.docx]

| **Compound** | ***P. cynomolgi* hypnozoite (pEC_50_)** | | | ***P. cynomolgi* schizont (pEC_50_)** | | | **Primary simian hepatocytes (pCC_50_)** | | |
| --- | --- | --- | --- | --- | --- | --- | --- | --- | --- |
|  | **NDO** | **NPI** | **XXJ** | **NDO** | **NPI** | **XXJ** | **NDO** | **NPI** | **XXJ** |
| **Cadralazine** | < 5.00 | < 5.00 | < 5.00 | < 5.00 | < 5.00 | < 5.00 | < 5.00 | < 5.00 | < 5.00 |
| **Poziotinib** | 5.67 | 5.95 | < 5.00 | < 5.00 | 5.96 | 5.51 | 5.60 | 5.82 | 5.46 |
| **Hydralazine** | < 5.00 | < 5.00 | < 5.00 | < 5.00 | < 5.00 | < 5.00 | < 5.00 | < 5.00 | < 5.00 |
| **Maduramicin** | 7.86 | 6.76 | 6.46 | 7.76 | 7.86 | 7.56 | 5.97 | 5.95 | 5.44 |

**Supplemental File 2**. Potency data (pEC_50_) for select ReFRAME hits against *P. cynomolgi* liver forms assayed at NITD in primary simian hepatocyte (PSH) lots NDO, NPI, XXJ infected with one batch of *P. cynomolgi* sporozoites. Cytotoxicity (pCC_50_) against PSH was measured using nuclei counts. Maduramicin is a positive control with activity against *P. cynomolgi* hypnozoites.
