# Supplementary material for: A drug repurposing approach reveals targetable epigenetic pathways in Plasmodium vivax hypnozoites
Source: eLife. 2025 Sep 30;13:RP98221. doi: 10.7554/eLife.98221 (PMC12483515; doi:10.7554/eLife.98221)
Supplement: Supplementary file 3. [file elife-98221-supp3.docx]

| ***P. vivax*** | **reads (#)** | **mapped reads (%)** | **mapped reads average genome coverage (x)** | **read methylation (%)** | **conversion rate (%)** | **average genome methylation (%)** | **percentage of cytosines meC/C >0.1 (%)** |
| --- | --- | --- | --- | --- | --- | --- | --- |
| Sample 1 | 42,081,431 | 2.40 | 1.90 | 0.36 | 99.79 | 0.28 | 0.78 |
| Sample 2 | 40,720,895 | 10.10 | 9.60 | 0.27 | 99.73 | 0.25 | 0.87 |
| Sample 3 | 33,682,926 | 10.10 | 9.72 | 0.60 | 99.70 | 0.57 | 2.00 |
| Sample 4 | 44,645,853 | 10.30 | 10.47 | 0.73 | 99.65 | 0.65 | 2.21 |
| **average** |  | **8.23** |  | **0.49** | **99.72** |  |  |
| **merged** | **161,141,105** |  | **31.69** |  |  | **0.49** | **0.89** |
|  |  |  |  |  |  |  |  |
| ***P. cynomolgi*** | **reads (#)** | **mapped reads (%)** | **mapped reads average genome coverage (x)** | **read methylation (%)** | **conversion rate (%)** | **average genome methylation (%)** | **percentage of cytosines meC/C >0.1 (%)** |
| Sample 1 | 52,657,816 | 1.40 | 2.08 | 0.48 | 99.73 | 0.28 | 1.00 |
| Sample 2 | 36,325,051 | 0.60 | 0.52 | 0.88 | 99.78 | 0.49 | 1.06 |
| Sample 3 | 39,109,832 | 1.80 | 4.00 | 0.39 | 99.75 | 0.27 | 1.06 |
| Sample 4 | 18,476,083 | 7.70 | 2.18 | 0.70 | 99.78 | 0.62 | 1.82 |
| **average** |  | **2.88** |  | **0.61** | **99.76** |  |  |
| **merged** | **146,568,782** |  | **8.68** |  |  | **0.39** | **1.38** |
|  |  |  |  |  |  |  |  |
| ***P. falciparum*** | **reads (#)** | **mapped reads (%)** | **mapped reads average genome coverage (x)** | **read methylation (%)** | **conversion rate (%)** | **average genome methylation (%)** | **percentage of cytosines meC/C >0.1 (%)** |
| Sample 1 | 44,871,635 | 77.40 | 115.29 | 0.42 | 99.84 | 0.58 | 0.37 |

**Supplemental File 3**. Summary statistics of read sets, percentage of mapped reads, read methylation levels, conversion rate and genome-wide methylation levels from bisulfite sequencing.
